# Screening of sensitive *in vivo* characteristics for early keratoconus diagnosis: a multicenter study

**DOI:** 10.3389/fbioe.2023.1158299

**Published:** 2023-08-04

**Authors:** Xuan Chen, Huazheng Cao, Yan Huo, Jiaxin Song, Haohan Zou, Jing Li, Jie Hou, Yan Wang

**Affiliations:** ^1^ School of Medicine, Nankai University, Tianjin, China; ^2^ Clinical College of Ophthalmology, Tianjin Medical University, Tianjin, China; ^3^ Tianjin Eye Hospital, Tianjin Key Lab of Ophthalmology and Visual Science, Tianjin Eye Institute, Nankai University Affiliated Eye Hospital, Tianjin, China; ^4^ Shanxi Eye Hospital, Xi’an People’s Hospital, Xi’an, Shanxi, China; ^5^ Jinan Mingshui Eye Hospital, Jinan, Shandong, China; ^6^ Nankai Eye Institute, Nankai University, Tianjin, China

**Keywords:** sensitive characteristics, *in vivo*, corneal biomechanics, corneal tomography, early keratoconus

## Abstract

**Purpose:** To analyze and compare sensitive *in vivo* characteristics for screening early keratoconus.

**Methods:** This multicenter, case-control study included 712 eyes, after matching for age and biomechanically corrected intraocular pressure, from three clinics in different cities. The keratoconus (*n* = 288), early keratoconus (*n* = 91), and normal cornea (*n* = 333) groups included eyes diagnosed with bilateral keratoconus, fellow eyes with relatively normal topography with unilateral keratoconus, and normal eyes before refractive surgery, respectively. After adjusting for central corneal thickness, differences *in vivo* characteristics were analyzed among the three groups. The *in vivo* characteristics were measured by Pentacam and Corvis ST. Fifty-four indices were evaluated to screen for a sensitive index for the detection of early keratoconus.

**Results:** Significant differences were observed in 26 of the 36 corneal biomechanical indeces between the early keratoconus and normal corneas. The area under the receiver operating characteristic curve of tomographic and biomechanical index, Belin/Ambrósio deviation, and Da in differentiating keratoconus from normal cornea was 1.000. Among the top five indeces of the area under the receiver operating characteristic curve for detecting early keratoconus, the corneal biomechanical-related index accounted for 80% (4/5), including A1 dArc length, highest concavity radius, A2 time, and tomographic and biomechanical index, of which the area under the receiver operating characteristic curve of A1 dArc length was 0.901.

**Conclusion:** A1 dArc length and several corneal biomechanical indices are highly sensitive for the detection of early keratoconus, even in the absence of topographic abnormalities. Ophthalmologists should focus on the clinical application of corneal biomechanics and combine corneal tomography for the timely and accurate detection of early keratoconus.

## 1 Introduction

Corneal refractive surgery decreases corneal stability, which may lead to postoperative ectasia if underlying pre-existing disease occurs, and result in disastrous refractive outcomes (Kim et al., 2019; Jabbour and Bower, 2021). Rigorous preoperative examinations are essential in corneal refractive surgery. Nevertheless, there is a paucity of reported cases in the literature, with a lack of clear risk factors for corneal ectasia (Duffey et al., 2008; Moshirfar et al., 2021). Hence, the preoperative screening of suitable patients remains a challenge for clinicians.

Keratoconus (KC) is one of the most common conditions and comprises a high proportion of patients who are unable to undergo corneal refractive surgery (Al-Amri, 2018). KC is an absolute contraindication for refractive surgery, with a reported incidence of 0.05%–0.23% (Rabinowitz, 1998). It is predominantly characterized by corneal thinning and forward protrusion, resulting in irregular astigmatism and severely reduced visual acuity (Santodomingo-Rubido et al., 2022). KC is one of the blindness diseases worldwide, and there is an urgent need to develop better methods for the accurate clinical diagnosis of early KC (EKC) to avoid corneal ectasia after refractive surgery, which may lead to vision loss that impacts the quality of life.

Currently, corneal topography and tomography are the main tools for diagnosing KC (Santodomingo-Rubido et al., 2022). However, as the morphology of EKC lacks evident abnormalities, relying on corneal tomography alone precludes an early diagnosis. Research on disease pathophysiology suggests that instability of corneal biomechanics changes corneal morphology, leading to corneal ectasia (Andreassen et al., 1980; Roberts and Dupps, 2014). Therefore, extensive efforts have been made to evaluate corneal biomechanical properties to detect keratoconus-like changes as early as possible. Differences in corneal biomechanics between normal corneas and KC have been reported in several studies. Corneal biomechanical changes play an important role in detecting EKC, especially in suspected cases or forme fruste keratoconus (FFKC) (Koh et al., 2020; Asroui et al., 2022). Nevertheless, detecting EKC remains difficult, as the detection ability remains weak in the early disease stage. Early detection of clinical measurements had been studied in previous studies (Shiga et al., 2021; Sedaghat et al., 2018), however, these findings are mixed, the reason for the inconsistent conclusions may be that the effect of age and intraocular pressure was not fully considered. An important feature of the present work is the investigation and analysis of the *in vivo* characteristics of normal corneas with age—and biomechanically corrected intraocular pressure-matched EKC to screen sensitive indices for diagnosing EKC.

## 2 Materials and methods

### 2.1 Participant inclusion and exclusion criteria

The study was approved by the ethics committee of Tianjin Eye Hospital (2022032) and performed in accordance with the principles of the Declaration of Helsinki. All participants signed an informed consent form to use their data for the analysis.

Patients enrolled from three clinics (Tianjin Medical University, Tianjin, China; Shanxi Eye Hospital, Shanxi, China; Jinan Mingshui Eye Hospital, Shandong, China) were divided into three groups according to the following criteria: 1) KC group: KC was diagnosed based on the diagnostic criteria of myopia and astigmatism history, corrected distance visual acuity (CDVA) < 20/20, abnormal corneal tomography (any of the following manifestations: asymmetric bow tie type - oblique radial axis, steep central or lower area, and Belin/Ambrósio Deviation (D) ≥ 3), and positive signs on slit lamp biomicroscopy (at least one of Vogt’s striae, Fleischer’s ring, Munson’s sign, or Rizutti’s sign) (Rabinowitz, 1998); 2) EKC group: unilateral KC comprising one eye diagnosed with KC and corneal tomography of the fellow eye was relatively normal (no asymmetrical bow tie type—oblique radial axis and no central or lower area steep), I–S of <1.4 D, KISA% index of < 60% (Rabinowitz et al., 1999; Steinberg et al., 2015a; Henriquez et al., 2020); and 3) Normal cornea (NC) group: patients whose refractive outcomes after SMILE have been demonstrated as safe without any complications throughout their at least 2 years of observation, with CDVA ≥ 20/20, normal slit-lamp biomicroscopy, and normal corneal tomography (none of the following manifestations: asymmetric bow tie type—skewed radial axis, steep central or lower area, and D ≥ 3), we select preoperative characteristics for analysis.

The exclusion criteria were ocular diseases other than ametropia and KC, history of other ocular operations, ocular trauma, and systemic diseases. All patients were required to stop wearing contact lenses (soft lenses for at least 2 weeks and hard lenses for at least 4 weeks) before assessment.

### 2.2 Ophthalmologic examinations

Each patient underwent routine ophthalmic examinations, including uncorrected distance visual acuity, CDVA, non-contact intraocular pressure, objective refraction, manifest refraction, slit lamp biomicroscopy, fundus examination, corneal tomography, and corneal biomechanical examinations. All the examinations were performed by the same technician.

Corneal tomography was examined using a three-dimensional anterior segment analysis system (Pentacam HR, Oculus, Wetzlar, Germany). Multiple images of the anterior segment were captured using a Scheimpflug camera to synthesize three-dimensional images of the anterior segment. All patients underwent measurements in a dark room. Patients were instructed to sit in front of the examination equipment in an upright posture and place their chin and forehead against the chin and forehead trays, respectively. After blinking several times, the patients were instructed to keep their eyes open and gaze at the flashing red dots during the rotation measurement of the equipment to automatically obtain images. Corneal biomechanical examinations were performed using the Corneal Visualization Scheimpflug Technology (Corvis ST, Oculus, Wetzlar, Germany). The ultra-high-speed Scheimpflug technology was scanned at a speed of 4,330 frames/s in the 8 mm horizontal range, and 140 images were acquired within 31 ms under the action of jet pulses. During the measurements, patients were instructed to place their chin and forehead against the chin and forehead trays, respectively, keep their eyes open, and gaze at the blue point after blinking several times. After the pressure-measuring head was aligned with the cornea, air was automatically ejected to obtain the dynamic response parameters of the cornea. The Pentacam and Corvis ST data were exported to CSV files using the original software of the equipment for analysis. The quality of all inspection results used for analysis was certified by experienced technicians.

### 2.3 Statistical analysis

Statistical analysis was performed using SPSS software (version 26.0; International Business Machines Corporation, Armonk, NY, United States). Continuous variables are expressed as mean ± standard deviation (minimum to maximum), and categorical variables are expressed as frequencies and percentages. The Kolmogorov–Smirnov test was used to test for normality. One-way analysis of variance was used to compare differences between groups of normally distributed variables, and a non-parametric test (Kruskal–Wallis test) was used to compare differences between groups of non-normally distributed variables. The chi-square test was used to compare differences between groups for categorical variables. Age and intraocular pressure (IOP) have effects on biomechanics, and central corneal thickness (CCT) has little effect on biomechanics (Huseynova et al., 2014; Valbon et al., 2014). Matching for age, biomechanically corrected intraocular pressure (bIOP), and adjusting for CCT using the general linear model, the Bonferroni test was used to analyze the differences *in vivo* characteristics between the two groups. Receiver operating characteristic (ROC) curves were used to evaluate and compare the 54 parameters (Supplementary Table S1). The cutoff value, sensitivity, and specificity were calculated when the differentiating ability was the best. Statistical significance was set at *p* < 0.05.

## 3 Results

### 3.1 Demographic and baseline characteristics

A total of 712 eyes were included after matching according to age and bIOP, with an average age of 22.92 ± 5.14 (range: 11–37) years. The KC, EKC, and NC groups comprised 288 eyes diagnosed with KC, 91 contralateral eyes with unilateral KC, and 333 eyes after refractive surgery 2 years, respectively. Details of the baseline characteristics are presented in Table 1.

**TABLE 1:** Baseline characteristics of the participants.

Parameter	NL (*n* = 333)	EKC (*n* = 91)	KC (*n* = 288)	*p*-value
Age	22.92 (19–26)	22.93 ± 4.91 (12–34)	22.95 (19.4–26.3)	0.250 ^0^
Sex (M/F)	211/122	64/27	179/109	0.360 ^0^
IOP (mmHg)	15.71(14.5–17)	14.57(13–16)	13.72(12.5–15)	<0.001 ^1^
				<0.001 ^2^
				0.007 ^3^
bIOP (mmHg)	15.42 (14.35–16.45)	15.39(14.2–16.5)	15.4 (14.3–16.5)	0.658 ^0^
CCT (µm)	552.45 ± 24.77 (479–601)	508.55 ± 36.2 (391–586)	467.62 ± 31.96 (375–575)	<0.001 ^1^
				<0.001 ^2^
				<0.001 ^3^
TCT (µm)	548.29 ± 24.94 (473–598)	501.44 ± 36.45 (379–583)	451.13 ± 32.9 (367–551)	<0.001 ^1^
				<0.001 ^2^
				<0.001 ^3^

Mean ± SD (range: minimum to maximum).

Kolmogorov–Smirnov test, One-way analysis of variance, Kruskal−Wallis test, and chi-square test was performed respectively.

^0^ *p*-value (NL, vs. EKC, vs. KC); ^1^ *p*-value (NL, vs. EKC); ^2^ *p*-value (NL, vs. KC); ^3^ *p*-value (KC, vs. EKC).

NL, normal cornea; EKC, early keratoconus; KC, keratoconus; IOP, intraocular pressure; bIOP, biomechanically corrected intraocular pressure; CCT, central corneal thickness; TCT, thinnest corneal thickness.

### 3.2 Differences in distribution of characteristics

Significant differences were observed in the corneal morphological parameters between the KC and NC groups before and after adjusting for CCT. Nonparametric testing revealed significant differences in 91.7% (33/36) of the corneal biomechanical parameters between the KC and NC groups, and 75% (27/36) were significantly different after adjusting for CCT.

Nonparametric testing revealed significant differences in corneal morphological parameters between the EKC and NC groups, except for Kmax (*p* = 0.384) and IHA (*p* = 0.613). After adjusting for CCT, no significant differences were identified except for Da (*p* < 0.001). Significant differences were observed in 72.2% (26/36) of the biomechanical parameters between the EKC and NC groups.

Analysis before and after adjusting for CCT revealed significant differences in corneal morphological parameters between the KC and EKC groups. Nonparametric testing revealed significant differences in 77.8% (28/36) of the corneal biomechanical parameters between the KC and EKC groups, whereas only 47.2% (17/36) of the parameters were significantly different after adjusting for CCT. Details are presented in Supplementary Table S2.

### 3.3 Evaluation of diagnostic ability for biomechanics and morphology

**FIGURE 1:**
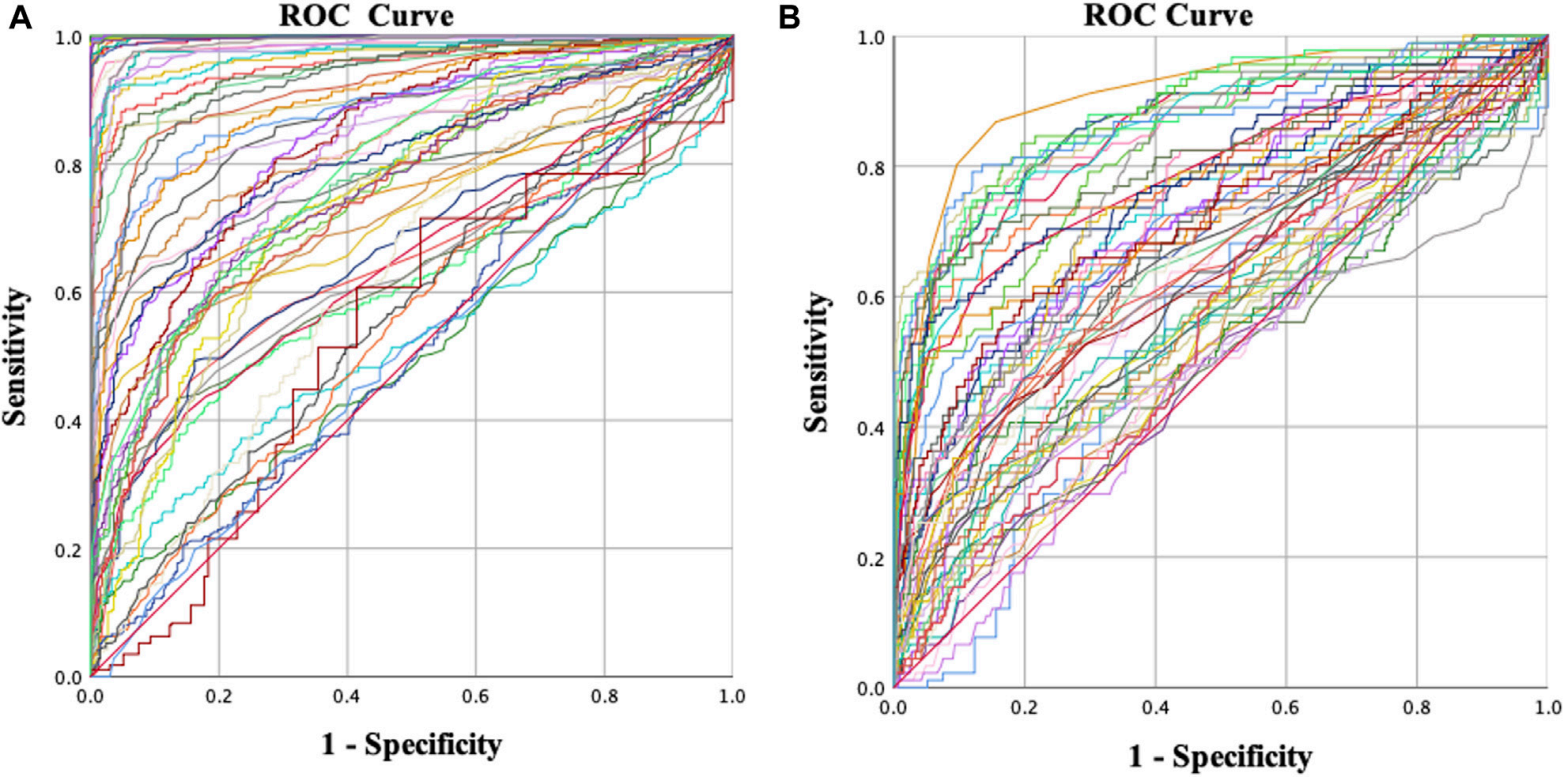
Receiver operating characteristic (ROC) curves for different groups. **(A)** Normal vs. keratoconus and **(B)** normal vs. early keratoconus.

The ROC curve analysis included 54 parameters, comprising 36 corneal biomechanical parameters and 18 corneal morphological parameters (Figure 1). When differentiating between KC and NC, the area under the ROC curve (AUROC) of the tomographic and biomechanical index (TBI), D, and Da was 1.000. The AUROC of most parameters was greater than 0.9 and had high sensitivity and specificity (Table 2).

**TABLE 2:** Results of receiver operating characteristic (ROC) curve analysis.

	KC vs. NL					EKC vs. NL				
	Parameter	AUROC	Sensitivity	Specificity	Cut off	Parameter	AUROC	Sensitivity	Specificity	Cut off
1	TBI	1.000	0.993	1.000	0.9375	A1 dArc Length	0.901	0.868	0.844	−0.0175
2	BAD-D	1.000	1.000	1.000	2.825	BAD-D	0.881	0.758	0.892	1.615
3	Da	1.000	0.997	0.997	1.81	HC Radius	0.879	0.835	0.805	6.9205
4	ISV	0.999	0.993	0.982	31.5	A2 Time	0.877	0.835	0.763	22.2025
5	Db	0.999	0.990	1.000	1.745	TBI	0.874	0.791	0.880	0.363
6	Dp	0.999	0.972	0.997	2.96	CBI	0.871	0.736	0.892	0.45
7	IHD	0.998	0.990	0.994	0.0275	Da	0.869	0.736	0.883	1.18
8	Df	0.998	0.972	0.994	2.665	Dt	0.865	0.758	0.856	0.415
9	CBI	0.997	0.979	0.018	0.659	TCT	0.865	0.758	0.856	523.5
10	IVA	0.997	0.969	0.988	0.255	CCT	0.849	0.670	0.907	522.5
11	Kmax	0.992	0.962	0.961	47.425	Max InverseRadius	0.849	0.780	0.799	0.1795
12	Dt	0.990	0.976	0.934	0.73	Dp	0.824	0.791	0.703	1.195
13	TCT	0.990	0.976	0.934	513.5	DA Ratio Max (1 mm)	0.817	0.648	0.931	1.616095
14	KI	0.987	0.958	0.976	1.075	A2 DeflectionVelocity	0.800	0.626	0.910	0.4095
15	Max InverseRadius	0.984	0.931	0.961	0.1945	Integrated Radius	0.790	0.560	0.949	9.4735
16	HC Radius	0.981	0.976	0.925	6.5835	A2 dArc Length	0.786	0.615	0.874	−0.0205
17	CCT	0.979	0.934	0.952	512.5	SSI 2	0.786	0.890	0.592	0.8815
18	Integrated Radius	0.975	0.920	0.973	9.607	A1 Deflection Length	0.747	0.736	0.664	2.2775
19	DA Ratio Max(1 mm)	0.969	0.906	0.964	1.628405	DA Ratio Max (2 mm)	0.732	0.538	0.931	4.820845
20	DA Ratio Max(2 mm)	0.956	0.882	0.952	4.876335	Whole Eye Movement Max [ms]	0.730	0.714	0.706	21.8855
21	SP A1	0.951	0.861	0.946	85.701	Db	0.728	0.538	0.841	0.735
22	K2 F	0.941	0.875	0.895	45.55	A2 Velocity	0.726	0.505	0.910	0.2955
23	SSI 2	0.931	0.837	0.895	0.8015	HC Deflection Length	0.726	0.824	0.562	6.709
24	Km F	0.921	0.840	0.910	44.85	HC Time	0.717	0.648	0.730	17.2035
25	CKI	0.907	0.858	0.955	1.015	A1 Time	0.716	0.780	0.586	7.4
26	SP HC	0.897	0.774	0.886	9.572	SP HC	0.714	0.593	0.805	10.224

ROC, receiver operating characteristic; AUROC, area under the ROC, curve; KC, keratoconus; EKC, early keratoconus; NL, normal cornea.

When distinguishing EKC from NC, corneal biomechanical-related parameters accounted for 80% (4/5) of the top five parameters in terms of discrimination ability, including A1 dArc length (AUROC = 0.901), highest concavity radius (AUROC = 0.879), A2 time (AUROC = 0.877), and TBI (AUROC = 0.874).

## 4 Discussion

To our knowledge, this study aimed to analyze and compare existing *in vivo* characteristics to screen sensitive indices to differentiate EKC and KC from NC. TBI, D, and Da had the best ability to distinguish between KC and NC; however, among the top five indices with the best ability to distinguish EKC from NC, four were related to corneal biomechanics, of which the force index A1 dArc length (AUROC = 0.901) was the best. This indicates the high sensitivity in corneal biomechanics for the diagnosis of EKC.

Detection of early KC, such as FFKC, in clinical practice is extremely difficult (Santodomingo-Rubido et al., 2022). KC is generally already in advanced stages once detected, resulting in irreversible vision loss. Patients with abnormal corneal morphology in both eyes and failure to meet the diagnostic criteria for KC are defined as KC suspect (KCS) (Klyce, 2009). It is challenging for refractive surgeons to diagnose patients with pre-clinical KC or a simple corneal tomography abnormality. TBI and the Corvis biomechanical index (CBI) have been proposed for the diagnosis of typical KC, but reports suggest that their ability to accurately diagnose EKC is relatively insufficient (Steinberg et al., 2015b; Herber et al., 2022). Therefore, this study aimed to identify a more sensitive index for diagnosing EKC.

The most important task was to control for confounding factors as much as possible, and age and bIOP were matched before inclusion of patients in this study because age and IOP had effects on biomechanics, that is, the effect of age and bIOP on the biomechanical properties was not significant difference among the three groups. Previous studies have found that corneal thickness has little influence on these parameters, although Corvis ST parameters correlate with CCT (Huseynova et al., 2014; Valbon et al., 2014). Furthermore, CCT was adjusted using a general linear model prior to analysis. No significant differences were observed in corneal morphological parameters between the EKC and NC groups, except Da (*p* < 0.001), but significant differences (*p* < 0.001) were identified in 26/36 biomechanical parameters. These results suggest that although corneal tomography remains relatively normal in EKC, the corneal biomechanics are altered. However, significant differences were observed in less than half of the biomechanical parameters between the KC and EKC groups, indicating that the biomechanical characteristics of EKC tended to be KC. Therefore, corneal biomechanical measurements should be performed routinely during refractive surgery.

According to the previous literature and directly reflecting the disease characteristics, we selected 54 indicators, including 36 biomechanic-related and 18 morphology-related parameters, which were included in the ROC curve analysis. Among the top five parameters that distinguished EKC from NC, corneal biomechanical-related parameters accounted for 80% (4/5), including the A1 dArc length, highest concavity (HC) radius, A2 time, and TBI. Among them, the AUROC of A1 dArc length was 0.901, and this index was the only indicator with excellent discrimination ability, which has not been previously reported. In terms of the safety of refractive surgery, this indicator may help clinicians accurately screen for EKC to avoid corneal ectasia after refractive surgery. Moreover, in terms of the disease itself, this indicator may enable early screening for KC and ensure timely intervention to avoid irreversible vision loss.

A1 dArc length represents the change in arc length of the anterior corneal surface within 3.5 mm on both sides of the corneal apex during the first applanation and the initial state. A softer cornea is associated with a larger deformation amplitude at the center of the anterior surface and a correspondingly larger value. As this indicator may be sensitive to focal changes in the early disease stage, other corneal biomechanical parameters were calculated based on a horizontal range of 8 mm (Lopes et al., 2017). This finding highlights the importance of considering sensitive biomechanical indices. HC radius refers to the radius of curvature of the corneal apex at the highest concavity. A softer cornea is associated with deeper collapse and a smaller radius of curvature. Considering the localization of the early stage of the lesion, this indicator has high sensitivity, and its ability to diagnose occult KC has been previously reported (Elham et al., 2017). The A2 time refers to the time to reach the second applanation, and the sensitivity of diagnosis has also been confirmed (Peris-Martínez et al., 2021). Considering the cumulative effect of this parameter, we avoided its use as a sensitive indicator in preoperative screening for refractive surgery. In accordance with our results, TBI has been reported to accurately detect corneal ectasia compared with other topographic, tomographic, and biomechanical parameters and has a high sensitivity for detecting eyes with normal topography in patients with very asymmetric ectasia (Ambrósio et al., 2017). TBI is based on morphology and biomechanics and reflects the importance of corneal biomechanics.

**FIGURE 2:**
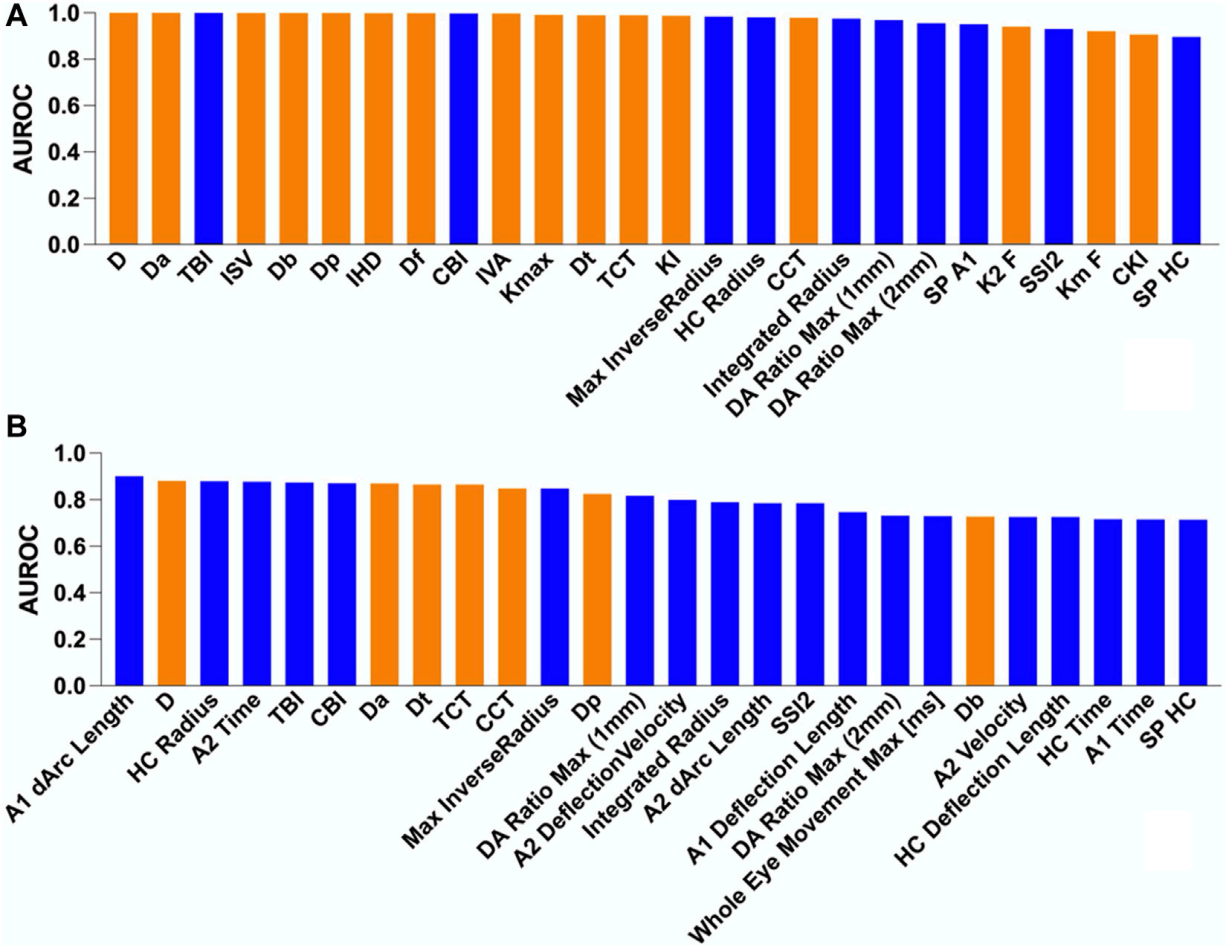
Comparison of the area under the receiver operating characteristic (AUROC) curve for different groups. The blue and orange columns indicate corneal biomechanic- and morphology-related parameters, respectively. **(A)** Normal vs. keratoconus and **(B)** normal vs. early keratoconus.

The ability to distinguish *in vivo* characteristics in the NC, EKC, and KC groups is presented in Figure 2. The orange columns in Figure 2A indicate the absolute advantage of corneal tomography in diagnosing typical KC. Most of the top ten indicators with the ability to distinguish KC from NC were corneal morphological indicators, with the exception of TBI (AUROC = 1.000) and CBI (AUROC = 0.997). These results suggest that corneal tomography of advanced KC is sufficient to provide reliable diagnostic evidence. TBI was first described by Ambrosio et al. based on corneal morphology and biomechanics (Ambrósio et al., 2017). A new parameter was introduced by cross-validation using the leave-one method to improve the discrimination ability between NC and KC in all other stages. In this study, TBI was the best index to distinguish KC from NC, which is consistent with previous reports (Ferreira-Mendes et al., 2019). CBI was introduced by Vinciguerra et al. by combining corneal dynamic response parameters (DA ratio, Vin, SP A1, and integrated radius) and ARTh using logistic regression (Vinciguerra et al., 2016a). In this study, we observed that CBI could accurately distinguish KC from NC, which is also consistent with previous reports (Langenbucher et al., 2021).

As shown in Figure 2B, the blue columns were more prominent, indicating that the corneal biomechanics were more sensitive in detecting EKC. The discrimination abilities of D, Dt, Da, CCT, and Dp were also good. However, considering that CCT was not matched when patients were included, the difference in distribution between the groups was significant. As D, Dt, Da, and Dp are affected by corneal thickness, the better discrimination ability of these parameters may be affected by corneal thickness. Indeed, in the early stages of the disease, corneal thickness does not decrease significantly, and corneal morphology does not exhibit obvious abnormalities. Therefore, corneal biomechanics is superior to corneal morphology for the detection of EKC. In the clinical screening of occult KC, more attention should be paid to changes in the corneal biomechanics.

Several *in vivo* characteristics (such as A1 dArc length, HC radius, A2 time, and TBI) exhibited good discrimination ability between EKC and NC (Table 2). Therefore, we calculated the corresponding cutoff value for the best discriminating ability and observed low sensitivity and specificity for most indicators. As proposed by Roberts and Dupps, this could be because the initial biomechanical changes in KC are focused rather than a uniform overall change (Roberts and Dupps, 2014). Hence, most corneal parameters have a large overlap between NC and KCS, thus reducing the sensitivity of detecting occult KC or simple corneal tomography abnormalities.

Owing to the complexity of viscoelastic biomechanical behavior and the inability to eliminate confounding factors of age, intraocular pressure and CCT (Huseynova et al., 2014; Valbon et al., 2014; Vinciguerra et al., 2016b), although we matched age and biomechanically intraocular pressure in this study, there are certain challenges in accurately measuring the real biomechanical properties *in vivo* (Huseynova et al., 2014). In addition, existing devices for measuring *in vivo* corneal characteristics are insensitive to spatial position and can only study surface deformation, precluding the characterization of internal corneal biomechanical behavior (De Stefano et al., 2020). Furthermore, selection bias was inevitable. Therefore, before early changes in the disease are identified, it is still necessary to combine corneal biomechanics with corneal tomography for clinical analysis.

In conclusion, this study demonstrated that TBI, D, and Da are the best indicators for diagnosing advanced KC and that corneal tomography is sufficient to provide a reliable diagnostic basis. The high sensitivity of corneal biomechanical index in the diagnosis of EKC, especially sensitive indicators such as A1 dArc length, suggests that ophthalmologists should pay more attention to the biomechanics when diagnosing EKC. The biomechanical properties of EKC are similar those of KC, indicating that corneal biomechanics should be combined with corneal tomography when screening for occult KC.

## Data Availability

The original contributions presented in the study are included in the article/Supplementary Material, further inquiries can be directed to the corresponding author.
