# Supplementary material for: Screening of sensitive in vivo characteristics for early keratoconus diagnosis: a multicenter study
Source: Front Bioeng Biotechnol. 2023 Aug 4;11:1158299. doi: 10.3389/fbioe.2023.1158299 (PMC10436515; doi:10.3389/fbioe.2023.1158299)
Supplement: Supplementary file 1 [file Table1.DOCX]

Supplementary Material

**Screening of Sensitive *In Vivo* Characteristics for Early Keratoconus Diagnosis: A Multicenter Study**

Xuan Chen, Huazheng Cao, Yan Huo, Jiaxin Song, Haohan Zou, Jing Li, Jie Hou, and Yan Wang^*^

*** Correspondence:** Yan Wang: wangyan7143@vip.sina.com

# Supplementary Tables

**Supplementary Table 1.** Corneal morphological and biomechanical parameters provided by the Pentacam and Corvis ST.

| **Parameters** | | **Parameters** | |
| --- | --- | --- | --- |
| CCT | Central corneal thickness | A1 and A2 deflection velocity | Deflection velocity at the first and second applanation |
| TCT | Thinnest corneal thickness | A1 and A2 deflection amp. | Deflection amplitude at the first and second applanation |
| D | Belin/Ambrósio deviation | A1 and A2 deflection area | Deflection area at the first and second applanation |
| Df | Deviation of front elevation difference map | HC time | Time to reach the highest concavity |
| Db | Deviation of back elevation difference map | HC radius | Radius at the highest concavity |
| Dp | Deviation of average pachymetric progression | HC dArc length | Arc length change from the initial state to the highest concavity |
| Dt | Deviation of minimum thickness | HC deflection amp. | Deflection amplitude at the highest concavity |
| Da | Deviation of ARTmax | HC deflection area | Deflection area at the highest concavity |
| Kmax | Maximum keratometry | HC deformation amp. | Deformation amplitude at the highest concavity |
| K1 F | Flat keratometry of corneal anterior surface | Max inverse radius | Maximum value of reverse concave radius |
| K2 F | Steep keratometry of corneal anterior surface | Peak dist. | Peak distance at the highest concavity |
| Km F | Mean keratometry of corneal anterior surface | Deflection amp. max. | Maximum deflection amplitude |
| ISV | Index of surface variance | Def. amp. max. | Maximum deformation amplitude |
| IVA | Index of vertical asymmetry | DA ratio max (1 mm/2 mm) | Corneal deformation ratio between corneal apex and corneal apex within 1 mm/2 mm |
| KI | Keratoconus index | Integrated radius | Integrated radius |
| CKI | Center keratoconus index | SP A1 | Stiffness-related parameter at the first applanation |
| IHA | Index of height asymmetry | SSI | Stress-strain index |
| IHD | Index of height decentration | SSI 2 | Stress-strain index was improved aiming to keratoconus |
| A1 and A2 time | Time to reach the first and second applanation | CBI | Corvis biomechanical index |
| A1 and A2 velocity | Velocity at the first and second applanation | TBI | Tomographic and biomechanical index |
| A1 and A2 dArc length | Arc length change from the initial state to first and second applanation |  |  |

**Supplementary Table 2.** Descriptive characteristics.

Mean ± SD (range minimum to maximum)

Kruskal−Wallis test and Bonferroni test were performed.

*P*-values were adjusted according to central corneal thickness.

^0^ P-value (NL vs. EKC vs. KC); ^1^ P-value (NL vs. EKC); ^2^ P-value (NL vs. KC); ^3^ P-value (KC vs. EKC)

NL = normal cornea; EKC = early keratoconus; KC = keratoconus.

| Parameter | NL（n = 333） | EKC（n = 91） | KC（n = 288） | P-Value | Adjust P-Value |
| --- | --- | --- | --- | --- | --- |
| BAD-D | 1.02 ± 0.53 (-0.77-2.37) | 2.29 ± 0.93 (0.07-4.59) | 8.73 ± 3.5 (3.28-21.69) | <0.001 ^1^  <0.001 ^2^  <0.001 ^3^ | 1.000 ^1^  <0.001 ^2^  <0.001 ^3^ |
| Df | 0.47 ± 0.96 (-1.73-3.71) | 1.06 ± 1.14 (-1.44-3.99) | 11.04 ± 6.76 (1.32-37.32) | 0.026 ^1^  <0.001 ^2^  <0.001 ^3^ | 0.064 ^1^  <0.001 ^2^  <0.001 ^3^ |
| Db | -0.04 ± 0.71 (-1.38-1.68) | 0.74 ± 1.02 (-1.33-3.53) | 8.79 ± 5.11 (0.73-28.47) | <0.001 ^1^  <0.001 ^2^  <0.001 ^3^ | 1.000 ^1^  <0.001 ^2^  <0.001 ^3^ |
| Dp | 0.85 ± 0.77 (-1.39-3.21) | 2.27 ± 1.38 (-0.56-6.41) | 8.26 ± 3.82 (1.81-23.31) | <0.001 ^1^  <0.001 ^2^  <0.001 ^3^ | 1.000 ^1^  <0.001 ^2^  <0.001 ^3^ |
| Dt | -0.27 ± 0.7 (-1.55-2.11) | 1.21 ± 1.27 (-1.19-6.45) | 3.06 ± 1.37 (-0.36-7.19) | <0.001 ^1^  <0.001 ^2^  <0.001 ^3^ | 0.266 ^1^  <0.001 ^2^  <0.001 ^3^ |
| Da | 0.56 ± 0.61 (-1.92-2.01) | 1.6 ± 0.7 (-0.61-3.07) | 2.99 ± 0.44 (1.58-3.91) | <0.001 ^1^  <0.001 ^2^  <0.001 ^3^ | <0.001 ^1^  <0.001 ^2^  <0.001 ^3^ |
| Kmax (D) | 44.32 ± 1.69 (39.7-49.8) | 44.87 ±1.66 (41.62-49.6) | 56.3 ± 6.54 (44.5-79.3) | 0.384 ^1^  <0.001 ^2^  <0.001 ^3^ | 0.060 ^1^  <0.001 ^2^  <0.001 ^3^ |
| ISV | 18.27 ± 5.29 (7-39) | 21.47 ± 6.63 (7-41) | 81.4 ± 31.14 (30-205) | 0.023 ^1^  <0.001 ^2^  <0.001 ^3^ | 0.098 ^1^  <0.001 ^2^  <0.001 ^3^ |
| IVA | 0.12 ± 0.05 (0.03-0.31) | 0.17 ± 0.09 (0.04-0.47) | 0.8 ± 0.38 (0.15-2.2) | 0.005 ^1^  <0.001 ^2^  <0.001 ^3^ | 1.000 ^1^  <0.001 ^2^  <0.001 ^3^ |
| KI | 1.03 ± 0.02 (0.95-1.11) | 1.04 ± 0.03 (0.98-1.11) | 1.21 ± 0.1 (1.01-1.63) | 0.022 ^1^  <0.001 ^2^  <0.001 ^3^ | 0.258 ^1^  <0.001 ^2^  <0.001 ^3^ |
| IHA | 6.63 ±5.16 (0-28.2) | 8.44 ± 7.46 (0.2-37.7) | 29.63 ± 23.57 (0-122.6) | 0.613 ^1^  <0.001 ^2^  <0.001 ^3^ | 1.000 ^1^  <0.001 ^2^  <0.001 ^3^ |
| IHD | 0.01 ± 0.006 (0.001-0.031) | 0.02 ± 0.01 (0.001-0.053) | 0.11 ± 0.06 (0.012-0.36) | 0.026 ^1^  <0.001 ^2^  <0.001 ^3^ | 0.582 ^1^  <0.001 ^2^  <0.001 ^3^ |
| A1 Time [ms] | 7.45 ± 0.25 (6.76-8.05) | 7.24 ± 0.35 (6.57-8.41) | 7.15 ± 0.23 (6.66-7.99) | <0.001 ^1^  <0.001 ^2^  0.060 ^3^ | 0.108 ^1^  1.000 ^2^  0.649 ^3^ |
| A1 Velocity [m/s] | 0.15 ± 0.01 (0.11-0.19) | 0.15 ± 0.02 (0.095-0.21) | 0.17 ± 0.03 (0.093-0.277) | 1.000 ^1^  <0.001 ^2^  <0.001 ^3^ | <0.001 ^1^  0.017 ^2^  0.030 ^3^ |
| A1 dArc Length [mm] | -0.019 ± 0.003 (-0.03-0.013) | -0.014 ± 0.004 (-0.031- -0.003) | -0.018 ± 0.005 (-0.045- -0.007) | <0.001 ^1^  <0.001 ^2^  <0.001 ^3^ | <0.001 ^1^  0.026 ^2^  <0.001 ^3^ |
| A1 Deflection Velocity [m/s] | 0.16 ± 0.02 (0.113-0.212) | 0.16 ± 0.03 (0.038-0.215) | 0.17 ± 0.04 (0.019-0.41) | 0.465 ^1^  <0.001 ^2^  <0.001 ^3^ | <0.001 ^1^  <0.001 ^2^  1.000 ^3^ |
| A1 Deflection Length [mm] | 2.32 ± 0.12 (1.84-2.76) | 2.16 ± 0.23 (1.01-2.62) | 2.29 ± 0.22 (1.23-2.87) | <0.001 ^1^  0.725 ^2^  <0.001 ^3^ | <0.001 ^1^  1.000 ^2^  <0.001 ^3^ |
| A1 Deflection Amp. [mm] | 0.096 ± 0.006 (0.053-0.116) | 0.09 ± 0.015 (0.046-0.114) | 0.106 ±0.016 (0.058-0.163) | 0.007 ^1^  <0.001 ^2^  <0.001 ^3^ | <0.001 ^1^  <0.001 ^2^  <0.001 ^3^ |
| A1 Deflection Area [mm^2] | 0.18 ± 0.02 (0.105-0.267) | 0.17 ± 0.03 (0.097-0.244) | 0.2 ± 0.04 (0.1-0.35) | 0.025 ^1^  <0.001 ^2^  <0.001 ^3^ | 0.014 ^1^  0.001 ^2^  <0.001 ^3^ |
| A2 Time [ms] | 22.48 ± 0.39 (21.18-23.51) | 21.81 ± 0.43 (20.66-23.22) | 21.89 ± 0.51 (18.34-22.96) | <0.001 ^1^  <0.001 ^2^  0.248 ^3^ | <0.001 ^1^  <0.001 ^2^  1.000 ^3^ |
| A2 Velocity [m/s] | -0.26 ± 0.03 (-0.36- -0.16) | -0.3 ± 0.54 (-0.47- -0.21) | -0.32 ± 0.59 (-0.48- -0.03) | <0.001 ^1^  <0.001 ^2^  <0.001 ^3^ | 0.002 ^1^  <0.001 ^2^  1.000 ^3^ |
| A2 dArc Length [mm] | -0.025 ±0.004 (-0.039- -0.01) | -0.02 ± 0.006 (-0.048- -0.006) | -0.02 ± 0.039 (-0.093-0.619) | <0.001 ^1^  <0.001 ^2^  <0.001 ^3^ | 0.664 ^1^  1.000 ^2^  1.000 ^3^ |
| A2 Deflection Velocity [m/s] | -0.34 ± 0.6 (-0.57- -0.12) | -0.49 ± 0.18 (-1.13- -0.24) | -0.53 ± 0.17 (-1.4 -0.21) | <0.001 ^1^  <0.001 ^2^  0.074 ^3^ | <0.001 ^1^  <0.001 ^2^  0.931 ^3^ |
| A2 Deflection Length [mm] | 2.85 ± 0.55 (1.719-6.412) | 2.78 ± 0.83 (0.637-6.047) | 2.67 ± 0.72 (0.355-6.177) | 0.137 ^1^  <0.001 ^2^  0.638 ^3^ | 1.000 ^1^  1.000 ^2^  1.000 ^3^ |
| A2 Deflection Amp. [mm] | 0.11 ± 0.01 (0.07-0.15) | 0.1 ± 0.01 (0.08-0.13) | 0.12 ± 0.07 (0.07-0.95) | 0.067 ^1^  <0.001 ^2^  <0.001 ^3^ | 0.805 ^1^  0.651 ^2^  0.053 ^3^ |
| A2 Deflection Area [mm^2] | 0.24 ± 0.04 (0.09-0.41) | 0.23 ± 0.05 (0.13-0.39) | 0.27 ± 0.27 (0.12-3.58) | 0.144 ^0^ | 1.000 ^1^  1.000 ^2^  0.619 ^3^ |
| HC Time [ms] | 17.44 ± 0.52 (15.21-18.61) | 17.06 ± 0.47 (15.85-18.13) | 16.99 ± 0.5 (15.5-18.5) | <0.001 ^1^  <0.001 ^2^  1.000 ^3^ | <0.001 ^1^  <0.001 ^2^  0.416 ^3^ |
| HC Radius [mm] | 7.46 ± 0.71 (5.68-10.08) | 6.27 ± 0.83 (3.7-9.22) | 5.17 ± 0.8 (3.22-7.64) | <0.001 ^1^  <0.001 ^2^  <0.001 ^3^ | <0.001 ^1^  <0.001 ^2^  <0.001 ^3^ |
| HC dArc Length [mm] | -0.14 ± 0.02 (-0.2- -0.08) | -0.12 ± 0.05 (-0.22-0.32) | -0.11 ± 0.04 (-0.23-0.4) | <0.001 ^1^  <0.001 ^2^  <0.001 ^3^ | 0.079 ^1^  0.002 ^2^  0.524 ^3^ |
| HC Deflection Length [mm] | 6.75 ± 0.4 (5.59-8.11) | 6.35 ± 0.56 (4.26-7.62) | 6.13 ± 0.47 (4.33-7.24) | <0.001 ^1^  <0.001 ^2^  0.001 ^3^ | <0.001 ^1^  <0.001 ^2^  <0.001 ^3^ |
| HC Deflection Amp. [mm] | 0.94 ± 0.09 (0.74-1.21) | 0.96 ± 0.12 (0.71-1.3) | 1.04 ±0.11 (0.78-1.42) | 0.530 ^1^  <0.001 ^2^  <0.001 ^3^ | <0.001 ^1^  0.056 ^2^  0.204 ^3^ |
| HC Deflection Area [mm^2] | 3.51 ± 0.46 (2.44-4.93) | 3.35 ± 0.6 (1.995-5.17) | 3.54 ± 0.52 (2.39-5.08) | 0.040 ^1^  1.000 ^2^  0.019 ^3^ | <0.001 ^1^  <0.001 ^2^  1.000 ^3^ |
| HC Deformation Amp. [mm] | 1.09 ± 0.09 (0.89-1.36) | 1.1 ± 0.11 (0.83-1.42) | 1.19 ± 0.12 (0.89-1.51) | 0.452 ^1^  <0.001 ^2^  <0.001 ^3^ | <0.001 ^1^  0.029 ^2^  0.295 ^3^ |
| Peak Dist [mm] | 5.17 ± 0.23 (4.49-5.77) | 5.01 ± 0.31 (4.27-5.9) | 4.98 ± 0.25 (4.27-5.73) | <0.001 ^1^  <0.001 ^2^  0.620 ^3^ | <0.001 ^1^  <0.001 ^2^  0.001 ^3^ |
| Deflection Amp. Max [mm] | 0.95 ± 0.09 (0.75-1.21) | 0.97 ± 0.13 (0.71-1.43) | 1.07 ± 0.15 (0.78-2.08) | 0.245 ^1^  <0.001 ^2^  <0.001 ^3^ | 0.006 ^1^  0.749 ^2^  0.161 ^3^ |
| Deflection Amp. Max [ms] | 16.62 ± 0.66 (14.33-17.96) | 16.23 ± 0.64 (14.47-17.63) | 16.39 ± 1.2 (11.43-32.52) | <0.001 ^1^  <0.001 ^2^  0.375 ^3^ | <0.001 ^1^  0.006 ^2^  1.000 ^3^ |
| Def. Amp. Max [mm] | 1.09 ± 0.09 (0.89-1.36) | 1.1 ± 0.11 (0.83-1.42) | 1.19 ± 0.12 (0.89-1.51) | 0.452 ^1^  <0.001 ^2^  <0.001 ^3^ | <0.001 ^1^  0.029 ^2^  0.295 ^3^ |
| Max InverseRadius [mm^-1] | 0.17 ± 0.02 (0.13-0.24) | 0.2 ± 0.05 (0.13-0.57) | 0.25 ± 0.05 (0.16-0.61) | <0.001 ^1^  <0.001 ^2^  <0.001 ^3^ | 0.002 ^1^  <0.001 ^2^  <0.001 ^3^ |
| DA Ratio Max (1mm) | 1.57 ± 0.04 (1.45-1.67) | 1.64 ± 0.08 (1.46-1.85) | 1.73 ± 0.11 (1.52-2.24) | <0.001 ^1^  <0.001 ^2^  <0.001 ^3^ | 0.017 ^1^  <0.001 ^2^  <0.001 ^3^ |
| DA Ratio Max (2mm) | 4.36 ± 0.34 (3.51-5.53) | 4.83 ± 0.61 (3.55-6.17) | 5.94 ± 1.19 (3.82-13.04) | <0.001 ^1^  <0.001 ^2^  <0.001 ^3^ | 0.655 ^1^  <0.001 ^2^  <0.001 ^3^ |
| Integrated Radius [mm^-1] | 8.23 ± 0.79 (6.33-10.37) | 9.6 ± 1.41 (6.06-12.75) | 12.44 ± 2.26 (7.2-19.85) | <0.001 ^1^  <0.001 ^2^  <0.001 ^3^ | 0.874 ^1^  <0.001 ^2^  <0.001 ^3^ |
| Whole Eye Movement Max [ms] | 22.32 ± 0.88 (20.79-31.87) | 21.74 ± 0.58 (20.57-23.09) | 21.54 ± 0.76 (17.55-24.21) | <0.001 ^1^  <0.001 ^2^  0.198 ^3^ | <0.001 ^1^  <0.001 ^2^  0.184 ^3^ |
| SP HC | 12.6 ± 2.57 (6.96-21.4) | 10.54 ± 3.61 (3.68-21) | 8.32 ± 2.31 (3.76-18.8) | <0.001 ^1^  <0.001 ^2^  <0.001 ^3^ | 0.305 ^1^  0.136 ^2^  1.000 ^3^ |
| SP A1 | 105.91 ± 14.35 (68.65-206.2) | 95.11 ± 21.34 (56.87-162.4) | 69.22 ± 16.43 (33.19-121.59) | <0.001 ^1^  <0.001 ^2^  <0.001 ^3^ | <0.001 ^1^  0.039 ^2^  <0.001 ^3^ |
| SSI | 0.86 ± 0.12 (0.56-1.26) | 0.85 ± 0.16 (0.58-1.31) | 0.7 ± 0.17 (0.36-2.23) | 1.000 ^1^  <0.001 ^2^  <0.001 ^3^ | 0.101 ^1^  0.003 ^2^  <0.001 ^3^ |
| SSI 2 | 0.91 ± 0.09 (0.66-1.18) | 0.8 ± 0.09 (0.55-1.04) | 0.71 ± 0.1 (0.42-1.04) | <0.001 ^1^  <0.001 ^2^  <0.001 ^3^ | <0.001 ^1^  <0.001 ^2^  <0.001 ^3^ |
| CBI | 0.21 ± 0.17 (0.001-0.85) | 0.64 ± 0.29 (0.04-0.995) | 0.96 ± 0.09 (0.352-1.000) | <0.001 ^1^  <0.001 ^2^  <0.001 ^3^ | <0.001 ^1^  <0.001 ^2^  <0.001 ^3^ |
| TBI | 0.17 ± 0.17 (0.000-0.916) | 0.668 ± 0.36 (0.000-1.000) | 0.997 ± 0.04 (0.42-1.000) | <0.001 ^1^  <0.001 ^2^  <0.001 ^3^ | <0.001 ^1^  <0.001 ^2^  <0.001 ^3^ |
